# Assessing social vulnerability to drought in South Africa: Policy implication for drought risk reduction

**DOI:** 10.4102/jamba.v9i1.326

**Published:** 2017-01-31

**Authors:** Fumiso Muyambo, Andries J. Jordaan, Yonas T. Bahta

**Affiliations:** 1 Disaster Management Training and Education Centre for Africa, University of the Free State, South Africa; 2 Department of Agricultural Economics, University of the Free State, South Africa

## Abstract

The aim of this article was to assess and identify social vulnerability of communal farmers to drought in the O.R. Tambo district in the Eastern Cape province of South Africa using a survey data and social vulnerability index (SoVI). Eleven social vulnerability indicators were identified using Bogardi, Birkman and Cardona conceptual framework. The result found that an SoVI estimated for O.R. Tambo district was very high with a Likert scale of 5 for cultural values and practices, security or safety, social networks, social dependence, preparedness strategies and psychological stress attributed for the high value of social vulnerability to drought. Indigenous knowledge and education had an SoVI value of 2, which was of low vulnerability, contributing positively to resilience to drought. The study also found that government involvement in drought risk reduction is limited; as a result, the study recommends that a national, provincial and district municipalities policy on drought risk reduction and mitigation should be developed.

## Introduction

Drought is not exceptional to South Africa alone. Approximately 60% of sub-Saharan Africa is considered to be susceptible to drought (Ngaka 2012). Ngaka (2012), Food and Agriculture Organization (FAO) (2013) and the Department of Rural Development and Land Reform (DRDLR 2013) concur that drought has been ranked a major concern in South Africa in terms of the total economic losses, as well as the number of people affected. Drought emergency in six provinces of South Africa affects 4 million (IRIN News 2004). O.R. Tambo district of Eastern Cape province has been reported as one of the worst drought affected districts (Gumenge 2010). According to the South African Weather Service, South Africa experienced a worst drought period in South African history since 1982. Prolonged droughts are a significant threat to the vulnerable communities of the region including O.R. Tambo district of South Africa. The Eastern Cape province including O.R. Tambo district is one of the six provinces that were declared as disaster areas in 1982–1983, 2003–2004, 2007–2008 and 2009–2010 by the National Disaster Management Centre (NDMC) (Gumenge 2010; NDMC 2016). In 2015–2016, Eastern Cape province was also declared as a drought disaster area by the Minister of Cooperative Governance and Traditional Affairs (SABC 2016). The 2015–2016 drought was a result of an extremely strong El Niño and is comparable with the 1933 and 1982 droughts in South Africa, with more than 2.7m households facing water shortages across the country (The Southern Times 2016). The most severe El Niño-induced drought in decades led to the official declarations of disaster in all but two provinces in terms of the disaster *Management Act*, 2002 (Agri SA 2016). The drought in South Africa costs farmers’ losses up to R10m (USD 689, 655.2) in 2015 (*R* = Rand, the South African unit of currency) (Bahta, Jordaan & Muyambo 2016).

The recurring drought events and their associated negative impacts on the people, such as the 2003–2004 and 2009–2010 droughts which were declared disasters by the then president, Thabo Mbeki, and the Minister of the Department of Water Affairs and Forestry, Buyelwa Sonjica (IRIN News 2004; Gumenge 2010). Drought risk is based on a combination of the frequency, severity, and spatial extent of drought and the degree to which a population is vulnerable to the effects of drought (UNISDR 2005). Be it enacted by the Parliament of the Republic of South Africa, section 1 act 57 of the *Disaster Management Act*, 2002 amended the definition of vulnerability by act 2015 No.16 ‘“vulnerability” defined as the conditions determined by physical, social, economic and environmental factors or processes, which increase the susceptibility of a community to the impact of hazards’. The amendment of the act is in line with the generally accepted definitions used in the international context and across sectors nationally so as to make the principal act simpler and easier to understand (Government Gazette 2015).

White and Howe (2002) argued that there is a realisation that effective natural hazard prevention and mitigation will need to address not only the hydrological–meteorological factors but also the economic and social factors which influence the greater society and reinforce the impact of hazardous events. Wilhite (2005) stated that social vulnerability to drought is increasing at an alarming rate in many parts of the world and South Africa and O.R. Tambo district is not an exception.

Social vulnerability emerges ‘as a concept for understanding what it is about the condition of people that enables a hazard to become a disaster’ (Adger 2006; Cutter 2003; Cutter, Boruff & Shirley 2003; Tapsell et al. 2005). Social vulnerability assessment, therefore, is the process by which the susceptibility of ‘elements at risk’ to drought hazard is estimated, and includes an analysis of the underlying causes of their vulnerability (Kafle & Murshed 2006). Dunning and Durden (2011) asserted that social vulnerability assessment defines the association present between social characteristics and vulnerability to drought, delineating the people at risk. Social vulnerability, according to Bogardi and Birkmann (2004), comprises exposure, susceptibility and coping capacity.

A key question to be addressed is whether there is need for the social vulnerability assessment of drought. Tapsell et al. (2010) asked whether there is any gain in such undertakings, because some authors have argued that all people are vulnerable. Indeed, it is just a subset of a full vulnerability assessment in drought risk assessment, but nevertheless very compelling and critical (Dunning & Durden 2013).

Social vulnerability assessments focus on the social aspects that make some people more vulnerable than others, though exposed to the same drought (Kafle & Murshed 2006). Some groups of people are more inclined than others to suffer damage or loss from drought. This variability may be influenced by socio-economic characteristics (Jordaan 2014; Kuhlicke et al. 2009; Wisner et al. 2004). Social vulnerability also provides a framework for isolating social causes for drought (Khoshnodifar, Sookhtanlo & Gholami 2012). Moreover, Birkmann (2006); Cutter et al. (2003); Fekete (2010); King and MacGregor (2000); White and Howe (2002); Wisner et al. (2004); Wongbusarakum and Loper (2011) contended that an analysis of risk management, resilience and adaptation is futile without the understanding of social vulnerability.

The contribution of social factors to vulnerability to drought was not a popular concept in South Africa in general and in O.R. Tambo district of Eastern Cape province in particular. Identifying and assessing the role of social vulnerability in drought risk reduction was a burning and a timely issue considering the current drought situation of South Africa to mitigate and reduce the risk of drought. Although all people living in drought areas are susceptible, the social impacts often mostly affect socially vulnerable people in society. The question is ‘Who are the vulnerable to drought in O.R. Tambo district, and what are the social characteristics or systems that make them vulnerable or resilient to drought?’ The need to understand these possible vulnerabilities motivated to carrying out this study so as to effectively suggest recommendations on vulnerability reduction measures to the farming community, policy and decision-makers. As defined by the *Disaster Management Amendment Act*, 2015 (No. 16) vulnerability incorporated physical, economic, environment and social factors that increase the susceptibility of a community to the impact of hazard, in this particular case drought. This study focuses on social aspects of vulnerability; however, it is worth noting that further research is needed to explore the physical, economic and environment aspects.

The main objective of the study was to assess and identify social vulnerability of communal farmers to drought in O.R. Tambo district in the Eastern Cape province. The finding of this result can apply not only from South Africa perspective but also from southern Africa perspective, considering similar socio-economic and demographic characteristics of the population. The research, which is reported in this article, is part of a more comprehensive research project on ‘Vulnerability, adaptation and coping with drought: The case of the commercial and subsistence extensive livestock sector in the Eastern Cape’ (Water Research Commission [WRC] 2014).

## Methodology

O.R. Tambo district was selected for the study mainly due to the recurring drought events and was reported as one of the most affected district in the Eastern Cape province of South Africa. Moreover, communal farming is the major agricultural practice in the district. The study used a survey data of 87 respondents from August to September 2014 and social vulnerability index (SoVI). Two separate interviews were held to collect data from farmers and extension officers during group discussions. The number of sample size of the study was influenced by the number of communal farmers who attended the workshops as well as their literacy levels.

**FIGURE 1:**
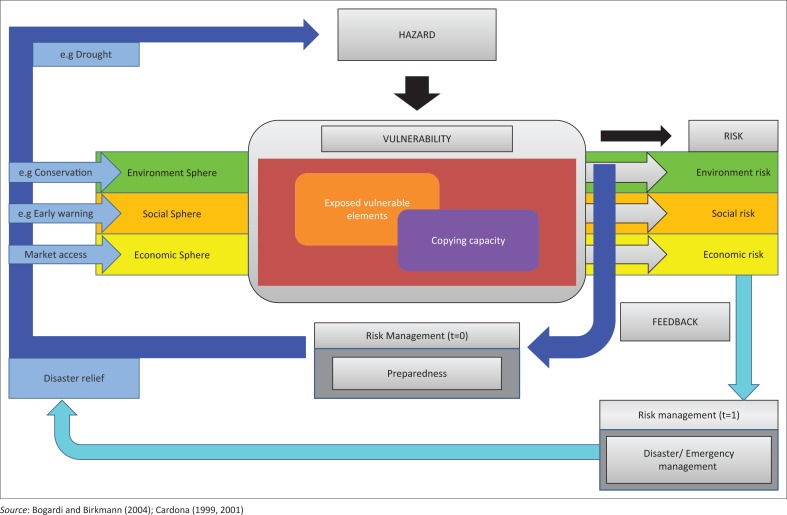
Conceptual framework for vulnerability.

A purposive sampling method was used to choose the type of respondents. A questionnaire was used to collect demographic data and socio-cultural characteristics of the farmers. In this study, the BBC framework by Bogardi, Birkmann and Cardona (Figure 1) was used to identify variables and interpret results. The term ‘BBC’ framework comes from the work on previous conceptual models by Bogardi and Birkman (2004) and Cardona (1999, 2001).

Eleven variables were identified for assessment of vulnerability in each household or farm with respect to drought according to the social vulnerability indicators (Table 1). The indicators were rated or classified according to a Likert scale that gave guidance in the estimation of the level of vulnerability: (0–1) very low vulnerability; (1.1–2) low vulnerability; (2–1.3) moderate vulnerability; (3.1–4) high vulnerability and (4.1–5) very high vulnerability.

**TABLE 1:** Selected social vulnerability indicators.

Indicators (variables)	Measure	Relationship with vulnerability	Source of data	Specification or description
Age	Above 60 years	Higher vulnerability	Survey and Stats SA	Number
Gender	Equality in decision-making – farming activities	Less decision-making – higher vulnerability	Survey	Male or female
Psychological stress	Stress influences vulnerability	Higher stress – higher vulnerability	Survey	Well-being of a farmer
Social dependence	Dependency ratio	Greater ratio – higher vulnerability	Stats SA	Social grants
Education	Formal education	More educated – less vulnerability	Survey and Stats SA	Formal education
Culture and practice	Influence vulnerability	Stronger cultural practice – greater vulnerabili-ty	Survey	Hold on to their livestock for honour and status – their wealth is locked up in their livestock Women are not allowed to enter an animal kraal – large numbers of goats are slaughtered for rituals A person is allowed to burn the veld after attending a ritual dance
Security or safety	Stock theft	Increasing stock theft – higher vulnerability	Survey	Farmers who feel insecure do not invest in their farming business and usually they suffer more from adverse drought impacts Stock theft Farm attacks
Social networks	Extent	More involvement in social network – lower vulnerabil-ity	Survey	Farmers’ organisations, churches, clubs, stokvels and family networks
External support	Level in drought mitigation and response	The greater external support – lower vulnerabili-ty	Survey	Government’s involvement in drought mitigation and response, interest in drought, training, funding, resource and information
Preparedness strategies	Prepare for drought	The more prepared – lower vulnerability	Survey	Availability of drought plans in the community and preparedness strategies for drought
Indigenous knowledge	Level	The higher indigenous knowledge	Survey	Traditional and cultural believes

Equal weights were assigned to indicators to calculate SoVI. Cutter et al. (2003) argued that there is no theoretical rationalisation for assigning different weights to indicate different levels of significance to individual factors’ contribution to social vulnerability. The indices were then summed up and divided by the total number of indicators to obtain the SoVI of the district. The SoVI will be calculated using the following mathematical equation:

$$V^{SOC=}\overset{11}{\underset{i=1}{\Sigma }}w_{i}^{soc}v_{i}^{soc}$$

$$V^{SOC}f(w_{1}^{soc}v_{1}^{soc},w_{2}^{soc}v_{2}^{soc},w_{3}^{soc}v_{3}^{soc},\ldots \;\ldots \;\ldots w_{11}^{soc}v_{11}^{soc}),$$ [Eqn 1]

where:

$$v_{1}^{soc}$$

= age,

$$v_{2}^{soc}$$

= gender participation,

$$v_{3}^{soc}$$

= psychological stress,

$$v_{4}^{soc}$$

= social dependence,

$$v_{5}^{soc}$$

= education level,

$$v_{6}^{soc}$$

= cultural values and practices,

$$v_{7}^{soc}$$

= security or safety,

$$v_{8}^{soc}$$

social networks, =

$$v_{9}^{soc}$$

= external support,

$$v_{10}^{soc}$$

= preparedness strategies,

$$v_{11}^{soc}$$

= indigenous knowledge,

$$w_{11}^{soc}$$

= equal weighting factor for all variables.

## Result and discussion

### Socio-economic characteristics of respondents

The respondents’ median age was 52 years above the national black South African median age of 24 years (StatsSA 2012), and the average age of 51 years shows that the younger generation was not involved in agricultural activities. The average farming experience was 13 years, although experience gained with age, farming need not be dominated by aged populations in any region, as this could have negative implications on the future of food production (Carino 2013). This study revealed that more men were involved in farming than women, with 71% of the respondents being men and 29% women. Khoshnodifar et al. (2012) found that wheat farmers in Mashhad County, Iran, were 84.5% men and 15.5% women.

The greater proportion of the respondents were married (74%), and widows (8%) comprise a very vulnerable group (Cutter et al. 2009) and may be more susceptible to drought impacts. Among the respondents, 26% did not have any schooling at all, which is higher than the district’s 17.3% illiteracy rate (StatsSA 2012).

Most farmers in this study practiced livestock farming, confirming that the Eastern Cape province is indeed a livestock farming region (ECDC 2014). The major categories of livestock they farm were cattle, sheep and goat. Most farmers lost their livestock through death, whereas some sold a portion to reduce their herd. There were reports of farmers who delayed in selling their livestock in preparation for imminent drought. Consequently, they later sold when their livestock could not fetch a good price. They had lost weight owing to poor quality and reduced quantity of feed as a result of dry conditions. One farmer reported that he increased his livestock during drought because he would buy them at reduced prices from other farmers.

### Social indicators of vulnerability to drought

Table 2 presents a summary of social indicators and their contributions to social vulnerability to drought. The results of the social vulnerability assessment of communal farmers to drought in the O.R. Tambo district municipality indicate a very high vulnerability score of 4.1 (Table 2). Out of 11 indicators, 6 indicators, namely, psychological stress, social dependence, cultural values and practices, security, social networks and preparedness strategies, contributed more significantly to social vulnerability to drought in O.R. Tambo district. These variables all scored 5 in the vulnerability measurement, which is rated as very high vulnerability.

**TABLE 2:** Estimation of social vulnerability index to drought in O.R. Tambo district.

Social Indicator	Findings	Index	Vulnerability
Age	23% ≥ 60 years	3	Moderate
Gender participation	62% responded gender affects agriculture-related deci-sion-making	4	High
Psychological stress	79% responded stress influences vulnerability	5	Very high
Social dependence	81% dependency ratio	5	Very high
Education levels	74% ≥ high school qualification	2	Low
Cultural values and practices	90% said cultural practices influence vulnerabili-ty	5	Very high
Security or safety	There was 25% increase in stock theft during drought	5	Very high
Social networks	18% responded social networks are involved in drought risk reduction	5	Very high
External support	22% responded government is involved in drought risk reduction	4	High
Preparedness strategies	9% indicated they prepare for drought	5	Very high
Indigenous knowledge	64% claimed to have indigenous knowledge on farming	2	Low vulnerability

**Total score**	-	45	-
**SoVI (total score ÷ no. of variables): 45 ÷ 11 = 4.1**	-	-	Very high

SoVI rating: (0–1) very low vulnerability; (1.1–2) low vulnerability; (2–1.3) moderate vulnerability; (3.1–4) high vulnerability and (4.1–5) very high vulnerability.

Gender and external support contribute highly to the social vulnerability of communal farmers to drought in O.R. Tambo district, which scored a 4. The huge imbalance in decision-making related to drought risk reduction influences social vulnerability to a great extent. The impacts affected the female gender, which is disadvantaged in decision-making, and also the whole farming enterprise. Livestock farming suffers more from lack of decision-making powers by women than crop farming does. This indicator is closely related to the cultural values and practices indicator, which was estimated at an index of 5, which is very high vulnerability. Women, according to tradition, cannot make decisions about reducing livestock, even if there is impending drought. Moreover, it was reported that they cannot enter a kraal because the cows will not reproduce. The cultural values and practices were also associated with the pride that Xhosa men have in their large livestock herds, which they will not easily reduce in preparation for an impending drought.

Age scored a 3. However, education level and indigenous knowledge scored low on vulnerability; hence, they are positively contributing to resilience to drought impacts. These two variables both scored 2 in the estimation of vulnerability, which is low vulnerability on the SoVI.

The result indicates that farmers perceived government’s involvement in drought risk reduction was below expectation, which was reiterated by extension officers. These findings coincide with other studies that revealed government as being limited in its involvement in drought risk reduction (Hassen 2008; Jordaan 2012; Ngaka 2012). The farmers indicated that the training programmes conducted by the extension officers in O.R. Tambo district do not focus on drought risk reduction, but on farming in general. There is no drought awareness or drought early warning issued to the farmers until the drought is already in progress. External support, therefore, contributes very little in helping farmers reduce their vulnerability to drought.

Social networks and preparedness strategies were initially considered under coping capacity. However, farmers are extremely exposed to drought impacts because they do not have any strategies to prepare for drought, neither do they have support from their social networks; hence, their coping capacity is severely undermined. The extension officers attributed the lack of involvement of social networks in drought issues to mere ignorance of their potential role. Generally, both the extension officers and farmers were surprised to learn that the involvement of clubs, churches and other community organisations in drought issues would increase the community’s resilience to its impacts. Kuhlicke et al. (2011) revealed that community networks and preparedness strategies were important factors in reducing social vulnerability to flooding.

The absence of operational and effective farmers’ association in the study area poses a challenge to farmers’ preparation and response to drought. The respondents argued that the associations operate at the top level. At the grassroots level, where the farmers need the support, they were said to be mostly absent or ineffective. Ntinga O.R. Tambo Development Agency (2014) confirms that sheep farming was well established in the communal farming areas of the O.R. Tambo district, although they need to be more organised in order to co-ordinate programmes in their communities. The Wool Growers’ Association solely depends on them. The contribution of lack of effective farmers’ institutions to vulnerability to drought was highlighted by Iglesias, Moneo and Quiroga (2007) who argued that participation of farmers in local institutions will lead to reduced vulnerability to drought.

Psychological stress contributes to a very high vulnerability situation. Although it is impossible to deduce from this study the reason why such a large proportion of the respondents held this perception, the issue of stress needs attention. As reported by Connor (2014), stress has been termed ‘the number one killer’ in the United States, and in this study, stress has been shown to contribute greatly to social vulnerability to drought. On 29 December 2015, in the Eastern Cape province of South Africa, one farmer, aged 34, a commercial cattle farmer, took his own life as the consequences of the worst drought had simply become too much for him (Chabalala 2016).

Dependence on government for social grants is very high in O.R. Tambo district (StatsSA 2012), and this increases farmers’ vulnerability to drought. Because drought mitigation is still more response-based than risk-reduction-based, farmers depend on government for assistance during and after drought. Dependency on social grants already indicates marginalisation and poverty (Adger et al. 2004), hence the inability to cope and recover from drought. It was argued that the dependency on social grants has created a dependency syndrome among locals.

The level of education, which was expected to contribute more to social vulnerability to drought in the O.R. Tambo district because of the district’s high illiteracy level, did not score high on the SoVI. Instead, education had a low vulnerability. The study revealed that the respondents perceived indigenous knowledge as contributing to the slight resiliency the farmers have towards drought. It has been argued that there is no community that is completely vulnerable (Adger et al. 2004; Birkmann 2006). The use of indigenous knowledge is relevant to a rural community that has high illiteracy levels. Where they cannot access information owing to illiteracy, indigenous agricultural knowledge will help them to cope with drought. This corroborates what UNEP (2008) claimed: indigenous knowledge is still integral among most African local or indigenous communities. The older people use indigenous knowledge to reduce the impact of disasters.

**FIGURE 2:**
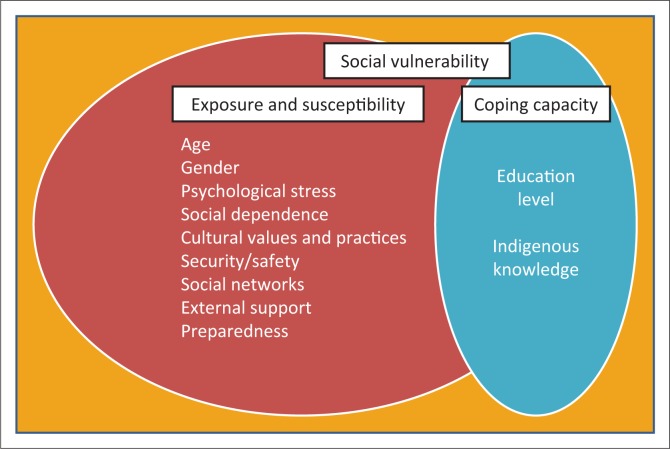
Summary of indicators using the BBC conceptual framework.

Figure 2 presents the different identified social indicators and their contribution to social vulnerability to drought. It is an extraction of the vulnerability segment from the BBC model showing the interaction of the components of vulnerability, exposure, susceptibility and coping capacity (Birkmann 2006). Indeed, this study, as argued by Fekete (2010), shows that no community is totally vulnerable. Whereas all indicators identified contributed to social vulnerability to drought in the study area, indigenous knowledge and education level fell within the coping capacity component, thereby contributing towards resilience to drought.

## Conclusion and recommendations

This study was undertaken to assess and identify the social vulnerability to drought of communal farmers in O.R Tambo district in the Eastern Cape province of South Africa using a survey data and SoVI. The result found that social vulnerability of communal farmers to drought in O.R. Tambo district is very high. Factors such as psychological stress, cultural values and practices, social dependence, lack of security, social networks and lack of preparedness strategies were the major contributors to social vulnerability to drought.

Government’s limited involvement in drought risk reduction, the age of the farmers and the imbalance of decision-making powers between the men and women also contributed to social vulnerability to drought. The cultural values and practices closely influenced gender dynamics. Based on the study, the following recommendations were drawn:

- A national policy on drought risk reduction should be developed for guidance. When drought occurs, it usually affects more districts or provinces. Therefore, a national policy on drought risk reduction should be developed which will guide provinces and district municipalities. The national policy should engage key role player for coordination and collaboration. There should be coordination among monitoring agencies in terms of reliable early warning data, communicated in a comprehensible way to decision-makers, effective farmer’s organisations (National African farmers union of South Africa and African Farmers Association of South Africa), Agri SA and private sectors. Collaboration with government departments at national and provincial level should also be strengthened. This includes collaboration with the Department of Agriculture, Forestry and Fisheries (DAFF) at national level, provincial Departments of Agriculture, NDMC and Provincial Disaster Management Centres (PDMC). Additionally, the provincial departments such as Provincial DAFF and PDMC should budget for risk reduction activities.
- Local representatives from farmers and extension services should participate in planning and implementing the drought plan.
- The DAFF should include programmes, which address drought mitigation (such as warning and preparedness) for guidance extension services. This could be achieved with the commitment, trust, devotion and willingness of main key player including politician, monitoring agencies, farmers’ organisation, private sectors, National and Provincial DAFF, NDMC and PDMC to take responsibility to implement drought policies and work efficiently towards achieving minimal drought impact.
- The security of livestock should be improved through the building and repairing of fences, as well as the reintroduction of rangers in the district. Security is a challenge for all communal farmers. Stock theft is a big problem, and communal land adjacent to the Lesotho border is particularly vulnerable – this is attributed by old fences. The study suggested that for the maintenance of old infrastructure such as fences, an infrastructure subsidy scheme should be provided as an initiative to build drought resilience by DAFF. Besides, as highlighted by the Minister of Department of Agriculture, Forestry and Fisheries of South Africa in 2015, in Annual Congress of National Wool Growers’ Association, ‘the South African merino develop the landrace breed (Dohne merino) – added versatility to the wool sheep gene pool and broadening the potential impact and range of the South Africa – need to continue intensively and expand in large scale’.
- The indigenous knowledge in the O.R. Tambo district should be compiled, documented and published for the young generation guidance.
